# Editorial: Novel Approaches in Microbiome Analyses and Data Visualization

**DOI:** 10.3389/fmicb.2018.02274

**Published:** 2018-09-27

**Authors:** Jessica Galloway-Peña, Michele Guindani

**Affiliations:** ^1^Department of Genomic Medicine, MD Anderson Cancer Center, Houston, TX, United States; ^2^Department of Infectious Diseases, Infection Control, and Employee Health, MD Anderson Cancer Center, Houston, TX, United States; ^3^Department of Statistics, University of California, Irvine, Irvine, CA, United States

**Keywords:** microbiome, data visualization, statistics, longitudinal data analysis, metagenomics, bioinformatics

Next generation sequencing technologies have allowed the study of microbial ecosystems at previously unseen depths. In both ecology and human biology, there is a pressing quest to advance our understanding of how microbial communities impact their host and their environment. In particular, the majority of microbiome studies are aimed at identifying specific microbial taxa, community profiles, genes, or metabolites which may be predictive of specific outcomes, functions, or disease states. However, due to the complexity of microbiome data, the statistical and computational analysis of these data present many challenges which may affect the validity of commonly employed methods. Therefore, despite the fact that microbiome and bioinformatic researchers often use widely accepted pipelines, the field remains wide open for improvement. In this Research Topic, a few researchers have responded to the task of reviewing or describing novel methodologies aimed at tackling the challenges of microbiome data and the respective metadata. Only with the development of improved statistical and computational models can one really hope to exploit microbiome based research to understand biological mechanisms, identify biomarkers of disease, or delineate microbial interactions with their environment.

The largest challenge investigators face in developing statistical approaches to study microbiome data is considering all of the constraints of microbiome data fully. Multiple researchers support regarding microbiome data as compositional, meaning the data are usually described as relative quantitative descriptions as parts of some whole, such as proportions or relative abundance. Of course, this view is also partial since important information may be lost when adopting a compositional perspective. However, intrinsic complications arise among commonly employed techniques if microbiome data are examined using a non-compositional paradigm. Gloor et al. reviews a number of recently proposed compositional data analyses methods for microbiome data, and provides some caveats against a naïve use of statistical models if the data are not treated as compositional.

One common problem among compositional microbiome data is that it is sparse and zero-inflated. This compositional bias leads to false positives as well as underpowered statistical associations when conducting multiple comparisons. A common strategy to handle excess zeros is to add a small number called a pseudo count. Kaul et al. propose a novel method (ANCOM-II) for handling zeros in microbiome data by first identifying the types of zeros in your data, then comparing the abundance of taxa relative to a background or reference value which is present in all specimens. Simulations of the authors' methodology show improved control for false discovery rate and higher statistical power compared to pseudo-counts. Another dilemma is that rare and low abundant taxa naturally exist among microbiomes. Karpinets et al. attempt to reduce the burden of filtering for the rare OTUs and overcome the difficulty of compositionality by treating the OTUs as qualitative variables. They explore the biological role of the rare low abundance OTUs and analyze them by using association networks (Anets) and show Anets have the potential to serve as a unsupervised methodology for linking rare OTUs to associations with environment or phenotypes.

Many methodologies naïvely separate themselves from the biologic aspect of the data inasmuch that these are complex interacting ecosystems with intertwined metabolic pathways, different rates of growth etc. Pinto et al. construct an ordinary differential equation (ODE) -based kinetic model incorporating microbial growth equations and metabolic interactions among bacteria using experimental data from gut microbe cultures. Their model accurately predicted bacterial abundance as well as metabolite consumption and production in a bioreactor experiment.

Furthermore, many approaches do not take into consideration phylogeny or relatedness of the organisms in order to make associations. Zhai et al. suggest a variance component selection scheme, or VC-lasso, for sparse and high-dimensional taxonomic data analysis. They disperse individual OTUs into clusters at phylogenetic levels, and translate the phylogenetic distance information to kernel matrices, where they treat the taxonomic clusters as multiple random effects in a variance component model. Similarly, Xiao et al. also develop a methodology for capturing clustered microbiome signals dependent on phylogeny. “glmmTree” is their novel prediction method based on a generalized linear mixed model, which captures clustered microbiome signals. In this framework, the effects of the bacterial taxa are modeled as random with the correlation structure dependent on a phylogenetic tree, whereas the effects of predictive variables are treated as fixed. Another conundrum is the concern that methodologies based on binning mapped sequences can still be riddled with error due to subpar databases. Currently OTU binning is the well accepted methodology, but group specific signatures can be just as important for biomarker discovery or disease association. Wang et al. recommend using K-mers which provides an alignment free method to characterize microbial communities.

There is a definite shortage of visualization or web based tools that support the integration of taxonomic and functional profiles. BURRITO, described by McNally et al. is a web based tool for interactive visualization of microbiome multi-omic data combined with taxonomic and functional information. BURRITO visualizes the taxonomic and functional compositions of multiple samples and underlines relationships between taxa and function. Baksi et al. present a web based framework called “TIME” (“Temporal Insights into Microbial Ecology”). TIME allows for predicting taxa that might have a higher influence on community structure in different conditions.

As substantial variability in microbiota communities can be seen across subjects, and across time, the improvement of longitudinal study design, and causal models is paramount to associate a dynamic ecosystem with complicated environmental and host factors. Many of the papers in this Research Topic offer methods which address different issues that arise when handling longitudinal data. The previously discussed web based application, TIME, was developed specifically to identify potential taxonomic markers from time series data (Baksi et al.). In this program, longitudinal time points, and respective metadata can be used to visualize temporal variations. Lee and Sison-Mangus developed a Bayesian semiparametric generalized linear regression model to investigate the effects of physical and biological variables the abundance of microbes. This model allows for borrowing information across OTUs, across samples and across time points. Shields-Cutler et al. introduce splinectomeR, an R package that uses smoothing splines to summarize categorical variables for hypothesis testing in longitudinal microbiome studies. Lastly, Wagner et al. propose the use of a bi-exponential function to summarize and compare diversity curves over time using hierarchical modeling. This approach accounts for repeated measures on each subject in order to compare and model alpha diversity indices over time.

Together, these original research articles and reviews emphasize the difficulties faced when analyzing microbiome data and the shortcomings of current statistical, computational and visualization tactics. Currently, many researchers perform all of their own coding and individual analyses without well-defined descriptions of their methods or sharing of analysis pipelines between laboratories. Thus, there is a pressing need for consistent and harmonious data analysis procedures. As a scientific community, microbiome researchers should not be content with “status quo” when it comes to widely accepted practices in microbiome analyses as they have many faults and limitations. In the modern era of data sharing and web-based tools scientists should be working together to compare results between sites and cohorts, improve current techniques, as well as validate methodologies. Only when the research community ensures that novel approaches hold up to independent validation across populations can we truly develop a microbiome analysis paradigm which allows for reliable reproducibility of findings across multiple institutions all over the world.

## Author contributions

All authors listed have made a substantial, direct and intellectual contribution to the work, and approved it for publication.

### Conflict of interest statement

The authors declare that the research was conducted in the absence of any commercial or financial relationships that could be construed as a potential conflict of interest.

